# Alpha-Synuclein Clearance through Inhibiting Akt/mTOR Pathway by Microfluidic Encapsulated Induced Conjunctival MSCs in a Parkinsonian Model

**DOI:** 10.30699/ijp.2022.553459.2890

**Published:** 2022-08-16

**Authors:** Meysam Forouzandeh, Mohammad Reza Bigdeli, Hossein Mostafavi, Samad Nadri, Mehdi Eskandari

**Affiliations:** 1 *Faculty of Life Sciences and Biotechnology, Shahid-Beheshti University, Tehran, Iran *; 2 *Institute for Cognitive and Brain Science, Shahid Beheshti University, Tehran, Iran*; 3 *Department of Physiology, School of Medicine, Zanjan University of Medical Sciences, Iran*; 4 *Cancer Gene Therapy Research Center, Zanjan University of Medical Sciences, Zanjan, Iran*; 5 *Department of Medical Nanotechnology, School of Medicine, Zanjan University of Medical Sciences, Zanjan, Iran*

**Keywords:** Alpha-synuclein, Mesenchymal stem cells, miR-149-5p, mTOR, Parkinson's disease

## Abstract

**Background & Objective:**

Parkinson's disease (PD) is a progressive neurodegenerative disorder in which the cause is attributed to the alpha-synuclein (α-Syn) accumulation due to the decreased rate of autophagy. Due to the many advantages, mesenchymal stem cells (MSCs), such as the secretion of neurotrophic factors, have been proposed for PD cell therapy. The present study, in continuation of the previous study, aimed to investigate the therapeutic effect of human-derived Conjunctival MSCs (CJ-MSCs) on the clearance of α-Syn by the microRNA-149(miR-149)/Akt/mTOR/ pathway.

**Methods:**

Stereotaxic 6-hydroxy dopamine (6-OHDA) was injected directly into the medial forebrain bundle (MFB) to induce Parkinson's disease. An apomorphine-induced rotation test was used to confirm the model establishment. CJ-MSCs were encapsulated in alginate microgel using a microfluidic system. The green fluorescent protein (GFP) labeled CJ-MSCs were encapsulated, and free cells were transplanted into the rats' right striatum. Behavioral and molecular analyses evaluated the potency of CJ-MSCs (encapsulated and free cells) in PD rats. Real-Time Quantitative Reverse Transcription PCR (qRT-PCR) was performed to investigate the expression of the miR-149-5p, Akt, mTOR, and α-Syn.

**Results:**

Our obtained results indicated that transplantation of CJ-MSCs leads to a decrease in the number of rotations while raising the balance and motor abilities. The gene expression evaluation showed a significant reduction in Akt, mTOR, and α-Syn mRNA levels and a significant increase in the level of miR-149-5p compared to the control group.

**Conclusion:**

It seems that CJ-MSCs can promote the degradation of intracellular α-Syn by miR-149-5p/Akt/mTOR pathway and improve rats' motor functions.

## Introduction

Parkinson's disease (PD) is the second most common neurodegenerative disorder after Alzheimer's; however, it is the most agile cause of neurological disability. PD is characterized both by motor and non-motor complications. Tremor, rigidity, bradykinesia, gait, and balance disorders are attributed to motor complications, while cognitive, behavioral, emotional, sleep, and autonomic problems are ascribed to non-motor problems (1). The most important pathological feature of PD is the loss of dopaminergic neurons following the formation of Lewy bodies (LB) in the dense part of the substantia nigra (SNpc), whose main components are alpha-synuclein, α-Syn, and ubiquitin (2). 

Alpha-Synuclein (α-Syn) is a 140-amino acids protein that binds to the membranes of synaptic vesicles. This protein does not turn into fibrils in the normal condition due to highly ordered and insoluble compounds, representing a dynamic equilibrium between soluble and membrane-anchored states (3). Following induction of PD by 6-Hydroxydopamine, the α-Syn level increased, which effectively expanded PD complications (4). In case of damage to lysosomal systems and ubiquitin-proteasome, fibril and Lewy body forms are observed (5). Although various mechanisms led to the dopaminergic neurons' degeneration following PD, the accumulation of misfolded α-Syn may be addressed as the most critical cause of cellular toxicity in PD pathology that finally leads to cell apoptosis (1). It has been recognized that malfunctioning of the natural destructive process, such as autophagy, may accumulate toxic proteins (6). Autophagy is defined as a cellular pathway involved in the destruction of damaged organelles and aggregated portions—the accumulation and expansion of misfolded α-Syn following impaired autophagy cause more stable cytotoxicity. Hence, increasing autophagy may be pursued as a potential and attractive therapeutic approach to reduce PD complications by intracellular degradation of α-Syn (7).

Mammalian target of rapamycin (mTOR) signaling is indicated to be involved in autophagy and apoptosis process regulating besides its essential role in cell development and tissue repair apoptosis (8). Previous studies have revealed that mTOR inhibition through some medications, including Rapamycin and Akt, could up-regulate the autophagy process in neurons (9). Animal studies have shown that this process finally protects dopaminergic neurons against MMP+ -induced cell death in a mouse model of PD and raises the autophagy rate in the C. elegans model (10).

Despite current therapeutic approaches, including restoration of dopaminergic tone and other pharmacological and surgical therapies, PD is still progressive. Therefore, identifying disease-modifying treatments seems to be required (11). The use of stem cell-based therapies as an effective and efficient treatment for PD has attracted much attention recently. 

The most significant limitations of cell transplantation are poor survival and vulnerability to neurodegeneration after transplantation attributed to neurotoxic factors and deficiency of trophic factors in the brain with PD (12). MSCs can migrate to damage DA neurons, enabling them to act as a targeted treatment to deliver effective factors in reducing PD symptoms (13). Mesenchymal stem cells (MSCs) are multipotent cells reported to differentiate into dopaminergic neurons after transfer to the brain with PD. MSCs presented their therapeutic effects through anti-inflammatory, immune modulation, cytokine secretion properties, and malfunction neurotrophic in the brain with PD (14).

Studies have shown that MSCs cells can secrete various factors, including microRNAs (miRs), and through these secretions, regulate gene expression and improve multiple disorders (15). miRs are small chains of non-coding RNAs that bind to the 3'-UTR region of mRNA to destroy or inhibit its target gene. Previous studies have shown that the level of miR-149-5p should decrease after cerebral ischemia (16) and Parkinson's disease (17) in the brain of rats, and its increase by miR mimic improves the complications caused by these neurodegenerative disorders. On the other hand, it has been determined that Akt, an upstream regulator of mTOR, which inhibits autophagy through elevating mTOR activity, is also one of the targets of miR-149. Increasing this miR's expression leads to increased autophagy by affecting the Akt/mTOR pathway (18).

Researchers have isolated a group of MSCs from the conjunctival epithelial cells (19, 20) that express dopamine-related genes with the potency to differentiate into dopaminergic lines. Besides, the high proliferation rate and long-term culture have turned them into a potential source for cell therapy (21). Despite all the advantages MSCs therapy presents, the difficulty of controlling the transplanted cells' fate and the use of immunosuppressive drugs to achieve maximum cell viability with the promotion of the remaining cells against the immune system are mentioned as the major challenges. Cell immobilization through biomaterials has been proposed as a practical approach to overcoming these problems. The stem cell microencapsulation method is an excellent method that provides a three-dimensional extracellular environment that enables easy control of cell migration, differentiation, and cellular parameters (22-24).

Our previous study investigated the effect of four groups of CJ-MSCs cells (encapsulated and non-encapsulated, inducible and non-inducible) on PD outcomes. It was found that injection of these cells increased TH and improved motor consequences of PD (25). Still, the possible molecular mechanism through which these cells can improve PD complications is not well understood. Hence, the autophagy process is disrupted, and α-Syn accumulation leads to damage to dopaminergic neurons during PD pathology. This study aimed to investigate the effect of encapsulated and non-encapsulated induced CJ-MSCs on the reduction of α-Syn level through the mTOR/Akt/miR-149 pathway.

## Material and Methods

**Animal Model**

In the present study, male Wistar rats weighing 220–270 g were used as the animal model for further investigation. The animals were kept under normal humidity, temperature, and light condition (12h light-dark cycle) for a week with the ad libitum available of water and food. The animal study was accommodated by the ethical committee for using laboratory animals at Zanjan university of medical sciences, Iran (IR-ZUMS.RE.1397.127).

**Fig. 1:**
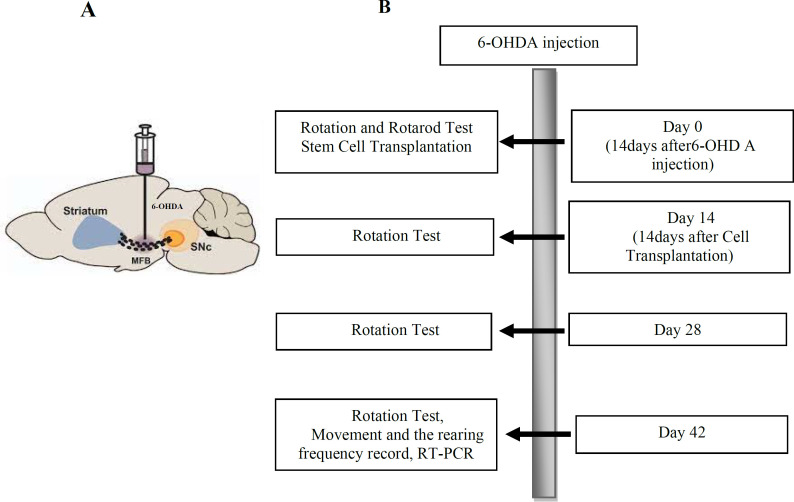
A schematic diagram of the 6-OHDA injection site (A) and timeline schematic diagram of the present study (B)

To establish PD models, briefly, the rats were anesthetized using ketamine and xylazine with the concentration of 100 and 10 mg/kg, respectively, and subsequently were fixed in a stereotaxic device (Stoelting, USA). A razor blade has been used to carve the rats' skull to determine the medial forebrain bundle (MFB) coordinates recruiting the Paxinos and Watson Atlas as AP: -4 mm bregma, ML: 1.8 mm from the midline, DV: 8.8 mm from the skull. A dental drill and a Hamilton syringe have employed to establish a small hole in the skull bone, and the injection of 6-OHDA (8µg/2µL normal saline containing 0.01% ascorbic acid, pH=5) into the area destroying the negro-striatal pathway, respectively (Figure 1A). 6-Hydroxy-dopamine can enter the dopaminergic nerve's terminal through a dopamine transporter. The mechanism in which PD is developed in animals is attributed to free radicals' production following the oxidization of injected 6-Hydroxydopamine. This phenomenon leads to dopaminergic neuronal death, interrupting mitochondrial function and oxidative stress (26). Five groups (n=40) of animals, each containing 8 Wistar rats, were investigated in the present study as follows: (1) the sham group: healthy rats that received only surgical stress; (2) the control group (PD Model): rats with Parkinson, that received no treatment; (3) vehicle group: rats with Parkinson, that treated with cell-free medium transplant in the right striatum; (4) iCJ-MSCs group: rats with Parkinson, that treated with induced CJ-MSCs (3×104 cells per 3μL in the right striatum); (5) the microfluidic encapsulated iCJ-MSCs group: rats with Parkinson, that treated with encapsulated induced CJ-MSCs (3×104 cells per 3μL in the right striatum) (Figure 1B). 

**Stem Cell Isolation and Characterization**

In the present study, a modified protocol has been recruited to isolate CJ-MSCs developed by Nadri et al. (21). Firstly, the CJ biopsy was treated with BSA and collagenase at the concentration of 40 and 4 mg/mL, respectively, followed by one-hour incubation in PBS (GIBCO-BRL, Grand Island, NY). The cell mixture was cultured in low glucose DMEM (GIBCO-BRL, Grand Island, NY) supplemented with 20% serum (GIBCO-BRL, Grand Island, NY) and 200 ng/mL basic-FGF (Peprotech, Rocky Hill, NJ) and incubated in a humidified chamber at 37°C with 5% CO2 for 14 days. In the final step, the cells were trypsinized (GIBCO) and expanded by two passages. In a previous study, the pluripotent potency of isolated stem cells was carried out through culture in different mediums, including osteogenic using (DMEM including 50 mg/mL ascorbic acid 2-phosphate (Sigma Chemical Co. St Louis, MO), 10 nM dexamethasone (Sigma Chemical Co.), 10 mM b-glycerophosphate (Sigma Chemical Co.); adipogenic using (DMEM supplemented with 50 mg/mL indomethacin (Sigma Chemical Co) and 100 nM dexamethasone (Sigma Chemical Co); chondrogenic using (DMEM supplemented with 10 ng/mL transforming growth factor-β3 (TGF-β3, Sigma Chemical Co), bone morphogenetic protein-6 (BMP-6), 10 -7M dexamethasone (Sigma Chemical Co.), 50 mg/mL ascorbate- 2-phosphate (Sigma ChemicalCo.), and 50 mg/mL insulin– transferring–selenium (ITS, GIBCO- BRL) for 21 days (25).

**Transduction and GFP Labeling of CJ-MSCs**

Toward GFP transduction to the isolated CJ-MSCs, a Lentivirus enriched medium was added to the complete media, followed by decanted to the cultured CJ-MSCs. Medium changing was performed after 24 hours. Puromycin antibiotic (2 mg/mL) was used to separate GFP labeled CJ-MSCs and expanded for 3–5 days in high glucose DMEM containing 10% FBS. GFP-labeled cells were detected using fluorescent microscopy. A neurogenic medium containing low glucose DMEM supplemented with 10 μM retinoic acid (RA, Sigma), 0.5 mM IBMX (Sigma Chemical Co), and 10μM forskolin (Sigma Chemical Co) was recruited for neural induction of the GFP-labeled CJ-MSCs (25).

**Microencapsulation of CJ-MSCs**

The soft lithography method has been used to fabricate microfluidic chips, according to Forouzandeh et al. (25). A su8-50 sensitive polymer was coated by the rotation speed of 3000 rpm on the silica-wafer as a solid phase for 5 minutes and was soft baked on a hot plate (65°C for 5 minutes and 95°C for 30 minutes). In the next step, the ultraviolet light with the wavelength of 330-430 nm was radiated to the coated silica-wafer. A developer solution was used to wafer immersion after soft baking, followed by nitrogen gas drying. To fabricate PDMS channels, a 1:10 w/w mixture of the crosslinker and Sylgard 184 (Dow Corning Corporation) was prepared and poured onto the fabricated masters. PDMS channels were formed through SU-8, and suitable holes were embedded to connect microtubes.

**Supplementary Figure 1:**
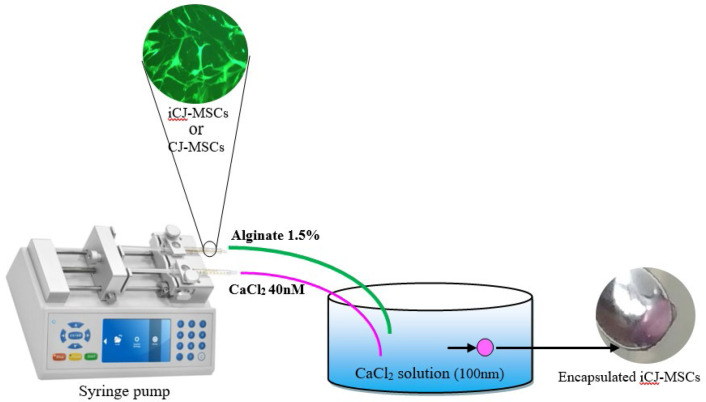
A schematic diagram of CJ-MSCs encapsulation using the microfluidic system. CaCl2 and alginate containing CJ-MSC were introduced separate inlets, and the encapsulated stem cells were harvested from the outlet.

**Supplementary Figure 2:**
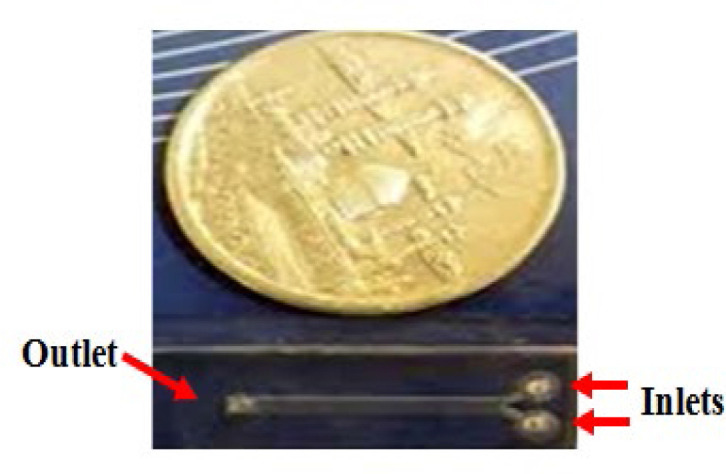
The designed microfluidic system for the present study. This system was a transparent chip with two inlets and one outlet.

Sodium alginate (Sigma, A2033) and calcium chloride (CaCl2, Merck, Germany) were used as the encapsulation and crosslinker materials, respectively. A total number of 3×104 passage-6 CJ-MSCs were cultured in DMEM medium containing 1.5% w/v, sodium alginate in non-adherent culture dishes. As shown in (Supplementary Figure 1), the alginate-containing cells and 40nM CaCl2 solutions were introduced to the two different inlets of the microfluidic chip (Supplementary Figure 2). The suitable flow rates for each solution were calculated based on the output microgel rigidity. Harvested microgel was incubated in a plate containing 100nM CaCl2 solution for 10 minutes to promote consistency. Finally, the appropriate encapsulated CJ-MSCs in alginate microgel were washed with PBS (25).

**Cell Transplantation**

Due to various advantages the stereotaxic method provided for cell transplantation, including short operation time and small surgery for local transplantation, which led to local anesthesia and concentrated transplanted cells to the desired area, CJ-MSCs were transplanted using this method. All rats were anesthetized with ketamine (100 mg/kg) and xylazine (10 mg/kg) and fixed in a stereotaxic device (Stoelting, USA). Free and encapsulated CJ-MSCs at the total number of 3×104/mL were transplanted through a Hamilton syringe into the rats' right striatum. Paxinos atlas was used to determine the right striatum area in which AP, 1.2 mm from the bregma, ML, +3 mm from the midline, DV 6 mm from the skull (25).

**Rotation Test**

In the present study, intraperitoneal injection of apomorphine at the concentration of 0.5 mg/kg was used to perform a rotation test. This test's underlying mechanism is based on the dopamine receptors' response of affected substantia nigra in animals to dopamine and dopamine agonists such as apomorphine. After apomorphine injection to the lesions, animals begin to rotate in the opposite direction. The total number of rotations is attributed to the intensity of the lesion. To perform the rotation test, rats were firstly adapted to the rotameter (Borj Sanat Azma RT-5300 Tehran, Iran) for 5 minutes. Next, the number of rotations to the opposite side of the lesion (left) was calculated for all rats using the rotameter following an intraperitoneal injection of 1 mg/kg of the apomorphine hydrochloride. We conducted this test on day 0 (two weeks after Parkinson's induction and just before cell transplantation), 14 (two weeks after cell transplantation), 28 (four weeks after cell transplantation), and 42 (six weeks after cell transplantation) (Figure 1B) (28). Data were expressed in the form of a complete body rotation every minute as the following equation:

Output rotations= Rotations in the lesion direction – rotations in the opposite direction of the lesion

**Movement and the Rearing Frequency Record**

The cognitive activities improvement of the rats was evaluated through an open field test after treatment. After adoption, inducted animals were placed in the center of the open field device (OPF, insight model open field EP 154C, Borj Sanat Azma RT-5300, Tehran, Iran) for 5 minutes to record their activity. The required parameters were evaluated as the movement frequency and the rearing frequency (28).

**RT-qPCR**

The expression of miR, Akt, α-Syn, and mTOR mRNA was assessed through Quantitative Real-Time PCR (RT-qPCR). Briefly, total RNA was extracted using Trizol, followed by quality and quantity determination using nanodrop (Themo Fisher, 2000). According to the manufacturer's instructions, mRNA cDNA Synthesis Kites (Stem Cell Technology Research Center BON209002, IRAN, and BON209002 Research Center, IRAN) have been recruited for cDNA synthesis. Syber green master mix (Amplicon, Denmark) has been employed to evaluate the gene expression using specific primers (Table 1) by RT-qPCR device (Life Technologies Corporation, USA). The mRNA levels were normalized to β-actin (for Akt,mTOR, and α-Syn) or Snord (for miRNAs) and quantified using 2∆∆Ct. 

**Statistical Analysis**

All statistics were reported as mean ± SD. One-Way ANOVA and Tukey post hoc test with the significance threshold of P-value<0.05 was used to analyze the data.

**Table 1:** Primer sequence for RT-qPCR

Primer sequences	Genes
**Forward: 5'-TCTGGCTCCGTGTCTTCACTCCC-3' ** **Common reverse primer in BON microRNA QPCR Master mix kit**	miR-149-5p
**Forward: ** **5ʹ-CATGAGGATCAGCTCGAACAGC-3** **Reverse: ** **5ʹACGGGCACATCAAGATAACGG-3ʹ**	Akt
**Forward: 5'-GGTTCCAAAACTAAGGAAGG -3'** **Reverse: 5'-CCTCCAACATTTGTCACTTG-3'**	α-Syn
**Forward: 5'-CGCGAACCTCAGGGCAA-3'** **Reverse: 5'-CTGGTTTCCTCATTCCGGCT-3' **	mTOR
**Forward: 5'-GCTCTGGCTCCTAGCACCAT -3' ** **Reverse: 5'-GCCACCGATCCACACAGAGT-3'**	β-actin

## Results

**CJ-MSCs Isolation and GFP-transduction**

In a previous study, the isolated conjunctival stem cells were cultured in osteogenic, adipogenic, and chondrogenic mediums to confirm the mesenchymal nature. The differentiation potency has been evaluated through alizarin red, oil red, and alcian blue staining. The presence of extracellular calcium, red-colored fat vacuoles, and purple color has been observed that imply stem cells differentiated into the osteogenic, adipogenic, and chondrogenic lineages, respectively (27). Microscopic observation of the green fluorescent protein (GFP) transduced CJ-MSCs has shown a green color a week after transduction. The cells demonstrated a nerve-like cell morphology due to exposure to the nerve induction medium (Figure 3).

**CJ-MSCs Encapsulation**

**Fig. 2:**
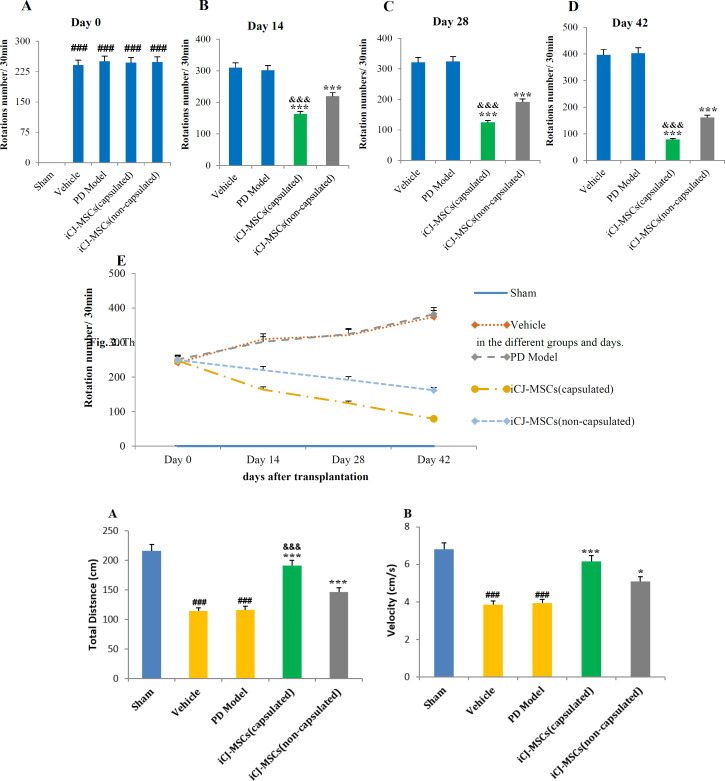
The bar and linear chart for the number of rotations induced by apomorphine in the different groups and days

A microfluidic system (Figure 2) with two inlets for sodium alginate containing CJ-MSCs and CaCl2 has been used to encapsulate CJ-MSCs. The channel diameter of the fabricated microchip was 300-600µm, in which the microgel was formed during the channels and extracted from the outlet to the dishes containing 100 nM CaCl2 (Figure 1). The flow rate of 1.5 and 3 mL/h has been used to introduce 1.5% w/v sodium alginate and a 40nM CaCl2 crosslinker. Figure 2 shows the experimental procedure of the CJ-MSCs encapsulation.

**Decreased Rotation Number Induced by Apomorphine following CJ-MSCs Transplantation**

**Fig. 3:**
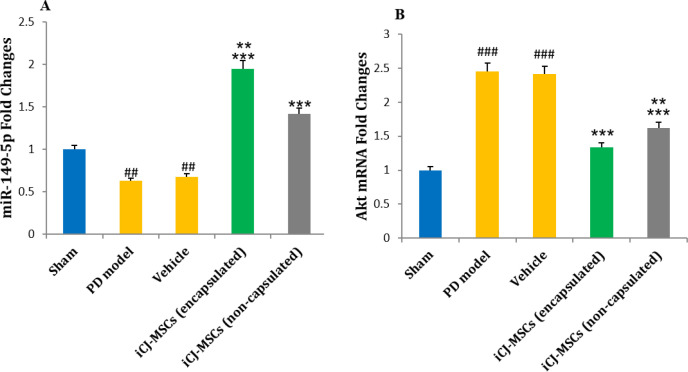
The traveled distance and mean velocity in the study groups

The obtained rotation test data have shown that PD induction leads to apomorphine-induced rotations to the opposite side of the lesion (left) in rats (Figure 2A). Encapsulated and non-encapsulated-CJ-MSCs transplanted rats have significantly reduced the number of rotations compared to the PD model group after 14 days (Figure 3B). Encapsulated- CJ-MSCs transplanted rats have also been shown more decreased rotation number compared to non-encapsulated-CJ-MSCs transplanted rats (Figure 2B-D). The number of rotations for the cell-treated groups showed a significant decrease on days 14, 28, and 42 compared to day 0. A significant difference was indicated for cell recipient groups on day 42 compared to 14 and 28 and on day 28 compared to day 14 were regarding the number of rotations (Figure 2E). 

The data obtained showed that injection of 6-OHDA toxin resulted in apomorphine-induced rotations to the lesion's opposite side (A). Fourteen days after CJ-MSCs transplantation, a significant decrease in the number of rotations was observed in the CJ-MSCs transplanted rats than in the model group (B). The data on days 28 and 42 indicated a decreasing trend in the number of rotations (C, D). It was significant in the encapsulated-CJ-MSCs transplanted rats compared to the non-capsulated CJ-MSCs. The number of rotations of the treated groups on day 42 significantly differed from those on days 28, 14, and 0. There was a significant difference between the number of rotations of day 28 with days 14 and 0 and day 14 with day 0(E).###*P*<0.001 vs. sham, ***P<0.001 vs. PD model and Vehicle, &&&*P*<0.001 vs. iCJ-MSCs (non-capsulated), $$$*P*<0.001 compared to all weeks of the same treated group. Values include mean ± standard deviation. (n = 8 per each group)

**Improved Motor Skills following CJ-MSCs Transplantation**:

Our investigations regarding the treated rats' movement ability revealed that PD induction has reduced the total distance (Figure 3A) and mean velocity (Figure 3B) of the rats compared to that of the sham group. However, both encapsulated and non-encapsulated CJ-MSCs transplanted PD rats have shown a significant increase in the total distance and the mean movement velocity relative to the PD model, in which encapsulated-CJ-MSCs transplanted rats showed more improvement compared to the non-encapsulated CJ-MSCs transplanted rats (Figure 3A and B).

Parkinson's induction decreased total distance (A) and velocity (B) in the PD model groups compared to the sham group. Transplantation of CJ-MSCs increased the total distance and velocity compared to the PD model group. It was also significantly increased in the encapsulated CJ-MSCs transplanted rats than in non-encapsulated CJ-MSCs transplanted rats (A, B).

###*P*<0.001 vs. sham, ***P<0.001 vs. PD model and Vehicle, *P<0.1 vs. PD model, Vehicle and iCJ-MSCs (capsulated), &&&*P*<0.001 vs. iCJ-MSCs (non-capsulated), $$$*P*<0.001 compared to all weeks of the same treated group. Values include mean ± standard deviation. (n = 8 per each group).

**Increase in miR-149-5p Level and Decrease in Akt following CJ-MSCs Transplantation**

**Fig. 4:**
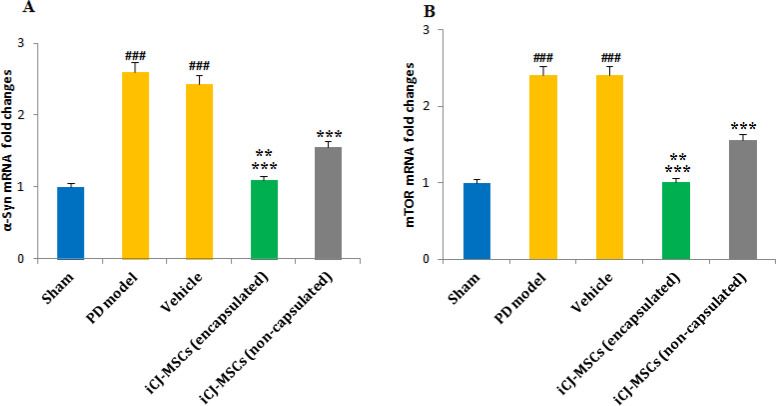
Changes in the levels of miR-149-5p (A) and Akt (B) in the studied groups

Gene analysis data showed that following the PD induction, the level of miR-149-5p was down-regulated, and a significant difference was revealed between the PD Model and sham groups. The increased regulation in the group that received encapsulated cells was higher than in the non-encapsulated. Administering both cell groups increased this miR level regulation compared to the PD Model group (Figure 4A). On the other hand, the examination of Akt mRNA level showed the up-regulation of Akt following PD induction and a significant increase in its level in the PD Model group compared to the sham group. In the groups that received encapsulated and non-encapsulated CJ-MSCs, a substantial decrease in Akt level was observed compared to the model group. Also, the level of Akt in the two cell receiving groups revealed a significant difference (Figure 4B).

The results show the down-regulation of miR and the up-regulation of Akt following the induction of PD. The injection of both cell groups caused a significant increase in miR levels and a significant decrease in Akt compared to the PD Model group. These changes were greater in the group receiving encapsulated cells than non-encapsulated cells. ###*P*<0.001 and ##*P*<0.01 vs. sham, ***P<0.001 vs. PD model and Vehicle, **P<0.01 vs. iCJ-MSCs (non-capsulated). Values include mean± standard deviation. (n = 8 per each group).

**Fig. 5:**
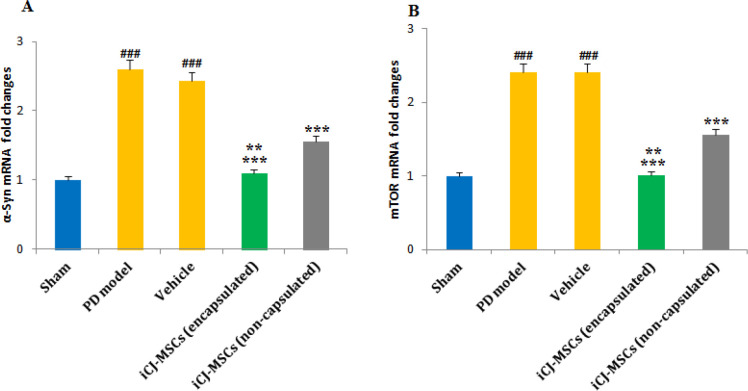
The changes in α-Syn and mTOR gene levels in different groups

**Decreased mTOR and α-Syn Levels in the CJ-MSCs Treated Rats**:

The results of RT-PCR showed that the α-Syn (Figure 5A) and mTOR (Figure 5B) mRNA levels had increased significantly following PD induction. Although Encapsulated and non-encapsulated CJ-MSCs transplantation reduced mTOR and α-Syn mRNA levels compared to the PD model, but encapsulated-CJ-MSCs showed to be more effective. And a significant difference in the level of these genes was observed in the group receiving encapsulated and non-capsulated cells (Figure 5A and B).

Following induction of Parkinson's, the levels of α-Syn (A) and mTOR(B) were significantly up-regulated compared to the sham group. CJ-MSCs transplantation decreased the regulation of α-Syn and mTOR mRNA levels. The reduction was more significant in the encapsulated CJ-MSCs transplanted rats than in non-encapsulated CJ-MSCs transplanted rats (A, B). ###*P*<0.001 vs. sham, ***P<0.001 vs. PD model and Vehicle, **P<0.01 vs. iCJ-MSCs (non-capsulated). Values include mean ±standard deviation. (n=8 per each group).

## Discussion

The results of our study showed that the transplantation of CJMSCs caused an increase in the level of miR-149-5, thus reducing the activity of mTOR by inhibiting Akt. Reduction of mTOR activity by activating autophagy causes alpha clearance and reduces its level in the brain of PD rats, thus improving motor complications caused by PD.

Despite the rapidly growing stem cell use for PD therapy, it encountered some challenges. Ethical concerns can be mentioned as an obstacle to embryonic stem cells' use (29). However, due to unique properties such as the ability to differentiate into dopaminergic neurons and stimulate the differentiation of host stem cells, MSCs shared a higher potential to prevent oxidative stress and neuronal death than other cells (30). Due to invasive methods and low isolated stem cells from bone marrow, appropriate new sources of MSCs are also needed (31). CJ-MSCs have been recently isolated from the conjunctival cells that can be used for PD treatment because of their ability to differentiate into dopaminergic cells (21, 25). Therefore, the easy manipulation of CJ-MSCs was a crucial factor in the previous study.

It was reported that α-Syn, the main constituent of LBs, regulates dopamine levels associated with dopamine deficiency in PD. α-Syn leads to dopamine reduction through interaction with TH (32). α-Syn can bind to the TH gene promoter and decrease its expression, decreasing the TH level (33). The accumulation of α-Syn has proven to be harmful to dopaminergic neurons. The toxic α-Syn may be transmitted from damaged cells to other adjacent cells, leading to the formation of LBs and disrupting the dopaminergic cells (34). The results of our previous study showed that TH expression increased after the injection of CJ-MSCs (25); on the other hand, in the present study, it was found that both encapsulated and free CJ-MSCs reduced the level of α-Syn. It can be concluded that decreasing the α-Syn level led to increased TH expression in PD rats' brains. Therefore, it seems that the activation of the pathways that reduce the level of α-Syn are effective in reducing the consequences of PD. As mentioned, an approach to reduce α-Syn is to induce its clearing by autophagy, and mTOR is considered a negative regulator for this pathway. According to previous studies, the mTOR signaling has increased during PD (35, 36).

On the other hand, it has been revealed that inhibiting mTOR signaling can up-regulate autophagy (37). It has been found that the increase in α-Syn expression is related to the decrease in mTOR signaling, and the decline in mTOR expression leads to an increase in the clearance of toxic and accumulated α-Syn, thus improving the outcomes of PD (38). It has been established that Akt is an upstream regulator and activator of mTOR. Its inhibition increases autophagy by reducing mTOR activity and leads to the reduction of α-Syn and improvement of PD complications (39).

Interestingly, levodopa, the commonly used drug, causes motor side effects (dyskinesia) through activating mTOR signaling in the mouse striatum. Rapamycin or its derivatives prevent the spread of levodopa-induced dyskinesia without exerting the anti-akinetic effect of levodopa on animal models (40,41). Ebrahim et al. have shown that MSC-derived exosomes can up-regulate autophagy by suppressing the mTOR pathway and their antifibrotic effect (42). In a similar study, MSC-derived exosomes were found to induce autophagy through AMPK/mTOR and Akt /mTOR, leading to a significant reduction in apoptosis and the volume of tissue damage (43). 

Vesicles secreted from MSCs can contain a variety of miRs, mRNA, and siRNAs, which upon entering the target cell, regulate the expression of genes, most of which are related to autophagy (44, 45). As mentioned, miR-149-5p leads to a decrease in Akt (mTOR activator), thus increasing the autophagy rate (18). It seems that the increase in the level of miR-149-5p after the injection of CJ-MSCs inhibits the Akt/mTOR pathway, clearing the accumulated intracellular α-Syand by increasing autophagy, thus improving the motor outcomes of PD. 

Studies have shown that cell encapsulation with microfluidic systems leads to neuronal proliferation and differentiation and increases viability (46). Alginate polymer is more favorable for cell encapsulation due to its biological simplicity and the provision of mild gelling conditions (47), leading to longer viability and maintenance of stem cell growth and division (48). Regarding the dopaminergic cells, it has been shown that cell encapsulation could vigorously promote cell survival, significantly increase TH levels, and improve animal behavioral and motor functions (49). In another study, cell survival was found to be increased up to 3.5-fold following the use of microencapsulation. After transplanting target cells into the striatum, TH levels increased, and motor function improved (50). Here in the present study, encapsulated-CJ-MSCs have been applied with more effective results regarding motor function, miR-149-5p, Akt, mTOR, and α-Syn, levels than the non-capsulated CJ-MSCs in PD inductive rats.

## Conclusion

According to our findings, it has been suggested that CJ-MSCs may increase the degradation of accumulated α-Syn within dopaminergic cells by decreasing mTOR activity through increasing autophagy. Thus, the TH expression has risen, and PD-induced motor complications have improved. Besides, cell encapsulation leads to the neuroprotective effect enhancement. Therefore, it may be possible to suggest CJ-MSCs as a source with therapeutic potential for PD and to encapsulate it as a mechanism to increase the neuroprotective effects.

## Funding

This research received no specific grant from any funding agency in the public, commercial, or not-for-profit sectors.

## Conflicts of Interest

The authors declared no conflict of interest.

## Ethics Approval

 The animals were tested according to the guidelines of the International Organization for Medical Science Working with Laboratory Animals. The protocol for working with animals was approved by the ethics committee of Zanjan University of Medical Sciences.

## Supplementary materials

**Supplementary Figure 3:**
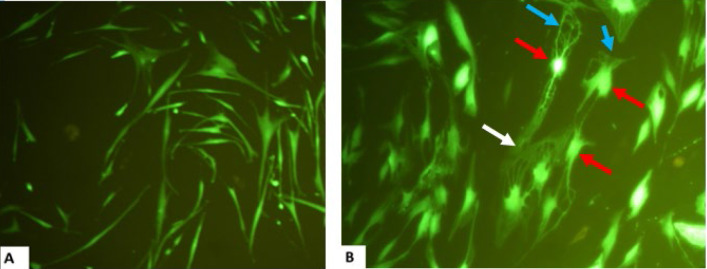
Obtained MSCs from the conjunctiva layer of the human eye (A), one week after exposure to the induction medium (B) And the appearance of a dense pyramidal cell body (red arrows), cell appendages (blue arrows), and neural network (white arrows) Magnification * 200.
